# Monomer Elution from Three Resin Composites at Two Different Time Interval Using High Performance Liquid Chromatography—An In-Vitro Study

**DOI:** 10.3390/polym13244395

**Published:** 2021-12-15

**Authors:** Krishnamachari Janani, Kavalipurapu Venkata Teja, Raghu Sandhya, Mohammad Khursheed Alam, Ruba K. Al-Qaisi, Deepti Shrivastava, Mohammed Odhayd Alnusayri, Zainab Ali Alkhalaf, Mohammed G. Sghaireen, Kumar Chandan Srivastava

**Affiliations:** 1 SRM Institute of Science and Technology, SRM Dental College, Chennai 600089, Tamil Nadu, India; jananik6@srmist.edu.in; 2 Department of Conservative Dentistry and Endodontics, Saveetha Dental College & Hospitals, Saveetha Institute of Medical & Technical Sciences, Saveetha University, Chennai 600077, Tamil Nadu, India; venkatatejak.sdc@saveetha.com; 3 Orthodontics, Department of Preventive Dentistry, College of Dentistry, Jouf University, Sakaka 72345, Saudi Arabia; mkalam@ju.edu.sa; 4 Prosthodontic Dentistry Department, Jordanian Royal Medical Services, Amman 962507, Jordan; rkqaisi@gmail.com; 5 Periodontics, Department of Preventive Dentistry, College of Dentistry, Jouf University, Sakaka 72345, Saudi Arabia; 6 Prosthodontic Dentistry Department, College of Dentistry, Jouf University, Sakaka 72345, Saudi Arabia; dr.moalnusayri@jodent.org (M.O.A.); dr.zainab.alkhalaf@jodent.com (Z.A.A.); dr.mohammed.sghaireen@jodent.org (M.G.S.); 7 Oral Medicine & Radiology, Department of Oral & Maxillofacial Surgery & Diagnostic Sciences, College of Dentistry, Jouf University, Sakaka 72345, Saudi Arabia

**Keywords:** dental restorations, Bis-GMA, bulk fill composites, conventional resin composites, high performance liquid chromatography, monomer release, residual monomer

## Abstract

Esthetics, improved colour stability and ease of contour have made photo-activated resin based restorative materials being widely used in routine dental clinical practice. Perhaps improper and inadequate polymerization of resin based composite material might lead to elution of monomer. Thus, the aim of the current study was to quantify the monomer elution from three resin composites. The intended analysis was made using high performance liquid chromatography (HPLC) at two different time periods. Three different materials that were investigated in the current study included Swiss Tech resin composite (Group A), Ceram X (Group B) and Beautifil Injectable composite (Group C). Ten cylindrical samples were fabricated in each study group. In 75% wt of ethanol, the samples were ingressed immediately and stored at room temperature. A 0.5 mL of the samples was assessed at pre-defined time intervals at 24 h and 7th day. Later, assessment of the samples was performed with HPLC and the data was analyzed using statistical test. Bisphenol A-glycidyl methacrylate (Bis-GMA), Triethylene glycol dimethacrylate (TEGDMA), 2-hydroxyethyl methacrylate (HEMA) and Urethane dimethacrylate (UDMA) were quantified in the samples. When analyzing the release monomer, it was found that at the end of 24 h Bis-GMA was eluted more in the injectable resin composite whereas, TEGDMA was eluted from Swiss Tech and Ceram X resin composites. At the end of the 7th day it was evident that Bis-GMA was eluted maximum in all the three resin composites. Thus, monomer release was found to be evident among all three resin composites and it is of utmost important to be assessed in routine clinical practice.

## 1. Introduction

There has been a great enthusiasm over recent decades in the use of tooth colored restoration. Resin composites are found to be potential alternative in replacing mercury containing amalgam restoration as a result of its esthetics and toxicological properties [1]. Resin matrix, inorganic fillers monomers, initiators and coupling agents constitute the composition of resin composites [2,3]. The most frequently used monomers are dimethacrylate such as bisphenol-A glycol dimethacrylate (Bis-GMA) and urethane dimethacrylate (UDMA) which are used since early 60s’. In recent years, to minimize the water absorption and polymerization shrinkage other composites were introduced [4]. Such composites are very viscous and therefore diluents such as triethylene glycol dimethacrylate (TEGDMA) were added [5].

During polymerization, conversion of monomer to polymer is termed as degree of conversion [6]. In other words, degree of conversion means the percentage of reacted C=C bond from the monomers present in polymeric matrices. Percentage of C=C bond calculated from the ratio of cured to uncured monomer is related to internal standard [7,8]. A hundred percent degree of conversion occurs when all monomer gets converted into polymer; however the conversion of all the monomer to polymer is never complete. The percentage of conversion usually ranges from 43–70%. The free monomer accounts for 10% elution from resin composite [9]. The degree of conversion depends upon the intrinsic factors such as concentration of photo initiator, chemical structure of the monomer and extrinsic factor such as polymerization conditions [10]. Factors which can influence the degree of conversion and are under the clinician’s control include the intensity of light from light curing unit, duration of irradiation light and the restorative material thickness [9].

The degree of conversion is inversely correlated with the unreacted monomer which in turn affects the solubility of resin composites. The solubility has an influence on the resistance to degradation in the oral environment [11]. Therefore, this property becomes critical as it affects the biocompatibility of the material at this stage. Decrease in the degree of conversions leads to increase in the elution of monomer into the oral environment and thus adversely affects the physical and mechanical property of the material [12].

The amount of eluting species ranges between 0.5 and 2% wt in water, 2–6% wt in 70% ethanol and 10% in methanol [13]. Various solvents employed to analyze the release of monomers include distilled water, saliva, ethanol, methanol and acetonitrile [14]. In the present study, ethanol was used as solvent which not only has the solubility parameter matching to that of Bis-GMA but it also matches to the oral environment for the detection of monomer elution. It was reported that unreacted monomers are said to be released when the ethanol penetrates the resin matrix and the space between the polymer chains expands [15].

Biocompatibility of the material gets affected when there is an increased release of monomer as the resin matrix consists of 20 to 40% of monomer. TEGDMA upon elution causes secondary caries by increasing the growth of cariogenic bacteria [16]. TEGDMA is reported to have cytotoxic effect as it can penetrate and react with intercellular molecules. Furthermore, it can creep into the membrane and can react with intracellular molecules. It is also identified as the primary component released from cured dental composites.

Bisphenol-A is reported to have a hormonal activity and has shown to affect the estrogen hormone leading to female infertility. Methacrylate monomers are very reactive in nature and in particular *in-vitro* research has shown that they may adversely interact with oral cells. Reactive oxygen species disturbs the redox homeostasis eventually disturbing the function of vital cells [17]. They are not only cytotoxic at high concentrations, but they also have been associated with genotoxicity [18]. Due to its chemical structure and hydrolic nature, Bis-GMA has shown a strong hemolytic potency.

Incremental layering technique enhances degree of conversion and increase in the percentage of monomer to polymer conversion when cured at the depth of 2mm. On the other hand, the disadvantages of this technique include the inclusion of voids or impurities between composite layers, bond failures between increments, insertion difficulty due to limited access in the small cavities and the requirement of additional time [19]. Unreacted monomer and other degradation products can be analyzed using high performance liquid chromatography (HPLC), gas chromatography, electrospray ionization and mass spectrometry (19). Ceram X (Dentsply, Konstanz, Germany) and Beautifil Injectable (Shofu Inc, Kyoto, Japan) are the two bulk fill resin composite which has been introduced recently.

Therefore, the aim of the study was to quantify the monomer elution of three resin composites. The analysis was made with the help of HPLC at two different time periods.

## 2. Materials and Methods

An in-vitro study was performed. The three different composites used in the current study were categorised into three study groups with a sample size of ten (n = 10) for each group. The solvent used in the study was ethanol. Table 1 lists the technical profiles of the three distinct composites employed in the investigation.

- Group A—Swiss Tech resin composite (Coltenewhaledent Pvt Ltd., Wazirpur, New Delhi, India)
- Group B—Ceram X (Dentsply Sirona, De-Trey-Straße 1, 78467 Konstanz, Germany)
- Group C—Beautifil Injectable (Shofu Inc., Fukuine, Higashiyama-ku, Kyoto, Japan)

### 2.1. Sample Preparation

Teflon moulds were used to make the samples, allowing for the creation of standardized cylindrical specimens (4 mm thickness and 5 mm diameter). The corresponding resin composite materials were placed into the moulds. For all study groups the resin composite was placed by incremental layering technique of 2mm thickness and cured. By applying pressure to the extra resin composite material, a glass slide was placed on top to assure flat surfaces. It also reduces the amount of oxygen that inhibits the polymerization reaction. The resin composite samples were polymerized for 20 s using a light-emitting diode unit in the standard curing mode, with an output wavelength ranging from 395–480 nm and 1000 mW/cm^2^ output irradiance. Following fabrication, ten samples from each group were immersed in a 75% wt ethanol/water solution as an extraction fluid and stored at room temperature in environmental chamber at 34 °C and 70% relative humidity. It is necessary to maintain the relative humidity in order to be equivalent to oral environment. 0.5 mL of ethanol sample was taken for HPLC analysis at predefined time intervals: 24 h (T1), and 7th day (T2). No other conditioning methods were taken for storing at 24 h and 7th day as it was only stored in ethanol solvent in order to assess the release of monomer.

### 2.2. High-Performance Liquid Chromatography (HPLC) Analysis

The samples were analyzed using HPLC (SHIMADZU, Model SPD 20A, Shimadzu Corporation, Nakagyo-ku, Kyoto, Japan). Liquid chromatographic grade ethanol and acetonitrile were used. The release of monomers was detected using reverse phase HPLC equipment. An EC 125/4 Nucleodur 100-5 C18 HPLC-Column was used to separate monomers. Ingredient of elution and composition of the mobile phase has been altered during the course of the chromatographic run. The composition of the mobile phase is increased gradually during the elution process. At a flow rate of 1 mL/min, the mobile phase was 80% acetonitrile and 20% ultrapure water, and detection was done at a wavelength of 254 nm. Loops with a capacity of 25 μL were injected. For the chromatographic peaks at the relevant retention time (RT) vs. monomer concentration, linear fits of the calibration curves were produced. The relationship between concentration and absorbance was displayed using calculated areas under the peak. The percentages of different polymers in each study group were calculated (Table 2).

### 2.3. Statistical Analysis

The collected data were analyzed with IBM SPSS statistics software 23.0 Version (IBM SPSS predictive analytics community, Armonk, NY, USA). One-way ANOVA with Tukey’s post-hoc test was used for the intergroup analysis for each time interval 24 h and 7th day. Intragroup paired comparison was also carried out with paired t-test to assess the monomer release.

## 3. Results

**Figure 1:**
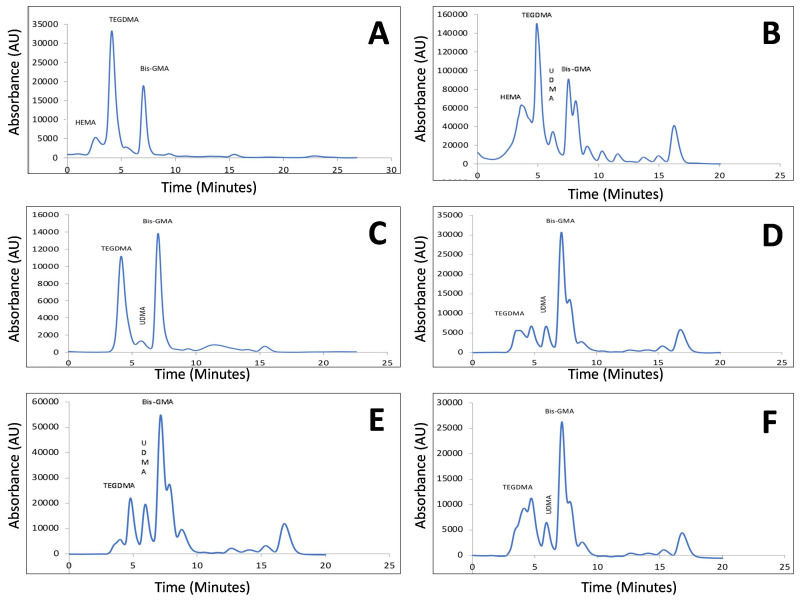
(**A**–**F**): Monomer Elution From Different Resin Composite At Two Different Time Interval Using High-Performance Liquid Chromatography (**A**) Ceram X composite Resin after 24 h; (**B**) Swiss Tech Resin Composite after 24 h; (**C**) Injectable Resin Composite after 24 h; (**D**) Ceram X Resin Composite after 7th day; (**E**) Swiss Tec after 7th day; (**F**) Injectable resin composite after 7th day.

Inter-group comparison after 24 h revealed higher overall monomer release from Swiss Tech resin composite followed by injectable composite and Ceram X. Evaluating the monomer release after 24 h from Group A, it was found that higher amount of TEGDMA was eluted, followed by Hydroxy ethyl methacrylate (HEMA), Bis-GMA, and UDMA. Within Group B, it was found that TEGDMA was eluted more than Bis-GMA and the least was UDMA. In case of Group C, Bis-GMA was reported to be eluted more than TEGDMA (Figure 1A–F).

After 7th day, in all three resin composites it was found that the most eluted monomer was Bis-GMA. The next eluted monomer was TEGDMA and least was found to be UDMA (Figure 1A–F). Paired t-test was used to quantify the monomer release as mentioned in the table (Table 3). Analysis of variance with Tukey’s post-hoc test revealed there was statistically significant difference in monomer release (Table 4 and Table 5).

## 4. Discussion

The release of monomers may have an impact on the material’s structural stability, biocompatibility, and wear rate. The most important factors for monomer elution are the chemistry of the solvent, size, chemical nature of the liberated components and the level of polymerization [20]. It can also be due to polymer matrix composition, filler particle type, content, resin porosity and homogeneity. Monomer release from the resin composite can be best evaluated by using HPLC than other methods such as gas chromatography, electrospray ionization, and mass spectroscopy [21]. HPLC, liquid chromatography-mass spectrometry (LC-MS), and gas chromatography (GC) are commonly used to identify the released components. For chemicals that can evaporate, it is advisable to utilize GC under normal circumstances. HPLC and LC-MS, on the other hand are better for samples with a high molecular weight, such as Bis-GMA and UDMA or that have a high potential to degrade when heated [22]. Additionally, the HPLC is faster, efficient, and accurate in the detection of monomer elution from the sample. Hence, in the current study HPLC was employed for the purpose of estimation of elution from the resin composites. The study aimed to assessed and compared monomer release from two packable and one injectable form of resin composite.

The majority of investigations employed artificial saliva, pure water, ethanol, methanol and acetonitrile as solvents. The amount of eluted monomer from a resin composite is said to be dependent upon the solvent [13]. Water and solvents such as ethanol, methanol and acetonitrile create a comparable environment in the oral cavity [3]. United States Federal Drug Administration has graded 75% ethanol–water solution as a clinical oral simulating liquid and it has been used in several studies [14]. In the present study, the rationale for using ethanol as a solvent was the ability of unreacted monomers to penetrate the matrix and widens the gap between polymer chains, thus allowing the soluble compounds to diffuse [23]. It can mimic and accelerate the typical degradation as expected clinically from the food and saliva through continual exposure [24]. Some chemicals in materials are remnants from the syntheses of raw materials and are not intentionally added during manufacture. However, this monomer can be discovered not only in ethanol-based eluates but also in water eluates.

Composite restoration in the oral cavity is said to release various components. These components are reported to cause cytotoxic, mutagenic, genotoxic and estrogenic effects [25,26]. The unpolymerized monomer has reported to reach the pulp thereby causing adverse pulpal reactions. The cytotoxicity was graded amongst all the monomer and was Bis-GMA was found to be more cytotoxic followed by UDMA, TEGDMA and HEMA [27,28]. Oxidative stress and depletion of cellular glutathione (GSH) occurs due to the toxicity of a number of dental materials to pulp cells [29]. The toxicity of methacrylates found in resin composites has been linked to GSH depletion and has been demonstrated to be mitigated by the promoter of GSH production and N-acetylcysteine. Bis-GMA causes toxicity to dental pulp cells, which gets exacerbated by the GSH synthesis inhibitor buthionine sulfoximine and mitigated by the GSH booster 2-oxothiazolidine-4- carboxylic acid [30]. Also, cellular stress gets created due to cytotoxicity of the restorative materials [31]. The appearance of free radicals, the initiation of apoptosis and tissue death are caused due to formation of cellular stress [32,33].

The size of the monomer molecule greatly influences the monomer release. Increased amount of monomer was reported to release with smaller size monomer molecule. Among the monomers, TEGDMA has a smaller molecular size and has hydrophilic property. Due to this property, TEGDMA gets liberated at a faster rate and was predominantly released from resin composite [34,35,36]. Due to enzymatic breakdown of monomer, higher amount of monomer gets liberated in the oral cavity when compared to that water. Studies have showed that TEGDMA and Bis-GMA were released in small quantities in water-based media compared to human saliva [37]. During polymerization, the monomers trapped in micropores in polymerized resin are more sensitive to elution than those trapped inside the microgel. If the volume of micropores is larger, then the solvent can penetrates the matrix more easily, thus cause widening of the gaps between polymer chains. A monomer can be eluted from a substance if it is soluble in the extraction medium. Thus, the concentration of eluted monomers and the rate at which they are eluted from the material are influenced not only by the percentage of unreacted monomers, but also by resin structure and monomer position within the polymer network. Tuna et al. [25] reported that the main monomer released was TEGDMA from all the samples whereas UDMA was released from only one group of material and Bis-GMA was not found in any samples. The degree of conversion of unreacted monomers and the regulation of its release using various procedures on various dental materials is a subject of ongoing research [38].

However, the results of the current study showed that at the end of 24 h in Swiss Tech resin composite group there was more release of TEGDMA followed by Bis-GMA, HEMA and UDMA. From injectable resin composite there was more elution of Bis-GMA followed by TEGDMA and UDMA. When we assess the monomer release after 7th day, it was evident that Bis-GMA was eluted more in all three resin composites followed by TEGDMA. Also, it was found that HEMA was eluted least to almost nil. The difference the monomer release amongst different resin composites can be due to various above-mentioned factors and also might be due to resin structure and the location of the monomer within the polymer network. The polymerized composite under the mylar matrix includes a surface layer rich in low molecular weight monomers. These smaller monomers are readily released after immersion in the extraction liquid. The removal of this layer by polishing could result in less TEGDMA being released from polished samples [39]. According to Yap et al. [40], any release that happens after 24 h of restoration is attributable to the hydrolysis process. The current investigation found that Bis-GMA eluted at a considerably higher percentage after 7th day. This finding is consistent with those of Olga Polydorou et al. and Komurcuoglu et al., who found that Bis-GMA was released more than TEGDMA [41,42].It could be owing to the difference in chemical characteristics and reactive potentials between Bis-GMA and TEGDMA, as Bis-GMA has a lower double bond conversion than TEGDMA [43]. Another reason could be the solution that was used to keep the samples in storage.

Ortengren et al. [2] examined the elution of monomers from resin-based materials with storage durations ranging from 4 h to 180 days, finding that the maximum monomer concentration was attained after seven days. Even after two weeks of ageing, several routinely used resin-based restorative materials continued to release considerable levels of mass into artificial saliva. Within 24 h, 85–100% of monomers are eluted, according to Ferracane and Condon [14]. Furthermore, more recent HPLC studies have shown that monomer elution for resin-based composites lasted longer than 24 h.

## 5. Strength, Limitations, and Future Directions

The strength of this present study is that assessment of monomer release was performed from three different resins composite, two packable composites and one injectable resin composite. There are very limited reports on monomer release from injectable resin composite. The quantification of monomer eluted was evaluated using HPLC, which is also considered as standardized method of assessment. However, in the present study, the evaluation of the monomer elution was assessed only at two-time interval. It is necessary to assess the release of monomer to identify till what time period monomer elution was present. In the present study ethanol was used as solvent, hence future studies can be concentrated on the usage of artificial saliva as a solvent in such a way it might be translatable to clinical situation.

## 6. Conclusions

Elution of monomer was found in all three composite resins at both time periods. Amongst all three composite Bis-GMA was released more followed by TEGDMA, UDMA and HEMA after 7th day. Therefore, it is essential to assess the amount of monomer release in composite resin and measures have to be taken to reduce the elution of monomer.

## Figures and Tables

**Table 1:** Technical profiles of the composites tested in the current study.

Composite	Manufacturer	Polymer	Filler Content % vol	Filler Content % wt
Swiss Tech (Group A)	Coltene	Bis-GMA UDMA, TEGDMA, HEMA	89	73
Ceram X (Group B)	Injectable Liechtenstein	Bis-GMA UDMA, TEGDMA, HEMA	62	81
Injectable (Group C)	Shofu	Bis-GMA UDMA, TEGDMA, HEMA	68.6	83.3

Note: Bis-GMA—dimethacrylate such as bisphenol-A glycol dimethacrylate; UDMA—urethane dimethacrylate; TEGDMA—Triethylene glycol dimethacrylate; HEMA—2-hydroxyethyl methacrylate.

**Table 2:** Monomer elution from three resins composite at different time interval.

Study Group	Polymers	24 h	7th Day
		% of Monomer Release	% of Monomer Release
Ceram X	HEMA	24.4	0
	TEGDMA	28.8	42.8
	UDMA	6.28	3.5
	Bis-GMA	12.8	49.0
Swiss Tech	HEMA	10.1	0
	TEGDMA	61.5	37.0
	UDMA	0.6	3.6
	Bis-GMA	24.25	57.3
Injectable	HEMA	0	0
	TEGDMA	42.1	38.7
	UDMA	5.3	3.5
	Bis-GMA	43.2	51.3

Note: Bis-GMA—dimethacrylate such as bisphenol-A glycol dimethacrylate; UDMA—urethane dimethacrylate; TEGDMA—Triethylene glycol dimethacrylate; HEMA—2-hydroxyethyl methacrylate.

**Table 3:** Intragroup comparative evaluation of the monomer elution from three resin composites at different time interval.

Study Group	Time Interval		*p* Value (Interpretation)
	24 h	7th Day	
Ceram X	10.01 ± 5.7	10.02 ± 5.7	*p* > 0.05; (NS)
Swiss Tech	13.02 ± 7.5	13.04 ± 7.5	*p* > 0.05; (NS)
Injectable	11.3 ± 6.5	13.3 ± 9.5	*p <* 0.000; (VHS)

Note: Results expressed in Mean ± Standard Deviation; NS—Not significant; VHS—Very High Significance.

**Table 4:** Inter group comparative evaluation of the monomer elution from three resin composite at different time interval.

Time Period	Groups	Sum of Squares	df	Mean Square	*p* Value
24 h	Between the groups	12,822.49	2	6411.246	0.0005 ***
	Within group	374,781.90	8254	45.406	
	Total	387,604.39	8256		
7th Day	Between the groups	12,809.68	2	6404.842	0.0005 ***
	Within group	374,781.90	8254	45.406	
	Total	387,591.59	8256		

Note: *** *p* value < 0.001; One-way ANOVA.

**Table 5:** Post-Hoc Intergroup comparative evaluation of the monomer elution from three resincomposite at different time interval.

Time Interval	Study Group	Comparison Study Group	*p* Value	95% Confidence Interval	
				Lower Bound	Upper Bound
Day 7	Ceram X	Swiss Tech	0.0005 ***	−3.457266	−2.601068
		Injectable	0.0005 ***	−1.729678	−0.845322
	Swiss Tech	Ceram X	0.0005 ***	2.601068	3.457266
		Injectable	0.0005 ***	1.327570	2.155763
	Injectable	Ceram X	0.0005 ***	0.845322	1.729678
		Swiss Tech	0.0005 ***	−2.155763	−1.327570
Day 24	Ceram X	Swiss Tech	0.0005 ***	−3.457266	−2.601068
		Injectable	0.0005 ***	−1.738011	−0.853656
	Swiss Tech	Ceram X	0.0005 ***	2.601068	3.457266
		Injectable	0.0005 ***	1.319237	2.147430
	Injectable	Ceram X	0.0005 ***	0.853656	1.738011
		Swiss Tech	0.0005 ***	−2.147430	−1.319237

Note: *** *p* value < 0.001; Tukey’s Post-Hoc analysis.

## Data Availability

The data set used in the current study will be made available on reasonable request.
